# Evaluation of Polytyramine Film and 6-Mercaptohexanol Self-Assembled Monolayers as the Immobilization Layers for a Capacitive DNA Sensor Chip: A Comparison

**DOI:** 10.3390/s21238149

**Published:** 2021-12-06

**Authors:** Ally Mahadhy, Bo Mattiasson, Eva StåhlWernersson, Martin Hedström

**Affiliations:** 1 Department of Biotechnology, Lund University, 22100 Lund, Sweden; allymahadhy@yahoo.com (A.M.); eva.stahl12@gmail.com (E.S.); martin.hedstrom@biotek.lu.se (M.H.); 2 Department of Molecular Biology and Biotechnology, University of Dar es Salaam, Dar es Salaam 16103, Tanzania; 3 CapSenze Biosystem AB, Värmö 5520, 26873 Billeberga, Sweden

**Keywords:** polytyramine, 6-mercaptohexanol, sensor chip, biosensor matrix, self-assembled monolayer, electropolymerization

## Abstract

The performance of a biosensor is associated with the properties of an immobilization layer on a sensor chip. In this study, gold sensor chips were modified with two different immobilization layers, polytyramine film and 6-mercaptohexanol self-assembled monolayer. The physical, electrochemical and analytical properties of polytyramine film and mercaptohexanol self-assembled monolayer modified gold sensor chips were studied and compared. The study was conducted using atomic force microscopy, cyclic voltammetry and a capacitive DNA-sensor system (CapSenze™ Biosystem). The results obtained by atomic force microscopy and cyclic voltammetry indicate that polytyramine film on the sensor chip surface possesses better insulating properties and provides more spaces for the immobilization of the capture probe than a mercaptohexanol self-assembled monolayer. A capacitive DNA sensor hosting a polytyramine single-stranded DNA-modified sensor chip displayed higher sensitivity and larger signal amplitude than that of a mercaptohexanol single-stranded DNA-modified sensor chip. The linearity responses for polytyramine single-stranded DNA- and mercaptohexanol single-stranded DNA-modified sensor chips were obtained at log concentration ranges, equivalent to 10^−12^ to 10^−8^ M and 10^−10^ to 10^−8^ M, with detection limits of 4.0 × 10^−13^ M and 7.0 × 10^−11^ M of target complementary single-stranded DNA, respectively. Mercaptohexanol single-stranded DNA- and polytyramine single-stranded DNA-modified sensor chips exhibited a notable selectivity at an elevated hybridization temperature of 50 °C, albeit the signal amplitudes due to the hybridization of the target complementary single-stranded DNA were reduced by almost 20% and less than 5%, respectively.

## 1. Introduction

For the design of a biosensor, how the sensor chip is designed is critical. The surface properties play an important role in the sensitivity, selectivity, and operational stability, i.e., the possible number of assays that can be performed on one sensor chip. Thus, the chemistry chosen when designing the sensor chip is of importance. This includes different parameters that can vary, such as the density of charges (negative or positive), and hydrophilic or hydrophobic material on the surface. The designing of the sensor chip includes the anchoring of the protection layer on the sensor chip. Then, it becomes important to select a suitable strategy for attaching the coating material so that no pinholes are present that may lead to poor analytical performance. Another important factor to consider when designing the sensor chip is the accessibility of the reactive groups on the coated sensor chip so that the capture agents can be immobilized.

The present study was carried out to optimize the properties of a biosensor for measuring the concentration of DNA fragments in a sample. The physical and chemical properties of a sensor chip surface determine the selectivity, sensitivity and stability of an electrochemical biosensor. Consequently, different immobilization layers have been used for the fabrication of biosensor gold sensor chip surfaces. The most reported immobilization layers for the fabrication of biosensor gold sensor chip surfaces are both conducting polymers, for example, polypyrrole [1,2,3,4], and non-conducting polymers, for example, polytyramine (Pty) [5,6,7,8,9,10]; as well as self-assembled monolayers (SAMs) of mercapto-containing organosilanes [11,12,13,14,15,16] and organosulfur compounds [17,18,19,20].

A major area of concern is the modification of the sensor chip with a layer that provides active groups for the immobilization of biomolecules in a defined manner, such that steric hindrance between molecules and their binding counterparts can be avoided. In capacitive biosensor measurements, the insulating properties of the immobilization layer are also an important factor. The surface of the sensor chip is designed to be highly insulated, in such a way that the capacitive biosensor system can be described as a simple model circuit, a resistor–capacitor (RC) circuit [21,22]. The RC model fits well with capacitive measurements using an exponential decay current curve [21]. Moreover, if the sensor chip is not sufficiently insulated, ions can move through the layer, causing a short circuit in the system, which leads to a deformed or omitted signal [22]. While polymers are characterized by their good insulation, making them useful for capacitive systems, SAMs are well known for their orderly self-organizing behavior, maximizing the availability of the active groups for the immobilization of bio-recognition molecules. Although both non-conducting polymers (polytyramine) and hydroxyl-terminated SAMs (6-mercaptohexanol) have been extensively used as sensor chip modification layers in capacitive biosensor systems [5,6,7,8,9,10], there are no comprehensive studies comparing the two layers to document/establish sound scientific evidence of the superiority/suitability of one layer over the other for capacitive DNA sensor measurements. Such a comprehensive comparison study will provide sound scientific guidance in selecting a sensor chip modification layer for capacitive biosensor systems, achieving ultrasensitive clinical assays. Usually, the sensor chip surface of a capacitive biosensor is very small, and the space is only enough for a small number of capture probes; therefore, maximizing the number of capture probes on the sensor chip surface while maintaining the capacitive (the RC model) system is vital for the ultrasensitivity of the biosensor.

In this report, results from an evaluation of two different chemistries and strategies—electropolymerization of tyramine (Pty film) and adsorption of 6-mercaptohexanol (MCH)-SAM—were used for the modification of gold sensor chip surfaces for capacitive DNA sensor analysis. The properties of the modified gold sensor chip surfaces were characterized by three different approaches: (i) electrochemical characterization, using cyclic voltammetry, CV (AUTOLAB, Utrecht, The Netherlands) and CapSenze™ Biosystem (CapSenze Biosystem AB, Billeberga, Sweden) measurements for determining insulating and hydrophobicity properties; (ii) topographical characterizations using atomic force microscopy, AFM, (XE-100 Park system, Suwon, Korea) for investigating the quality and uniformity of the sensor surface layer directly by imaging the surface and (iii) analytical characterization using CapSenze™ Biosystem when selectivity, sensitivity and re-usability were also determined and compared. The aim was to determine the immobilization layer that offers the best insulating properties and sufficient active sites for the immobilization of capture probes, as well as excellent analytical characteristics.

## 2. Materials and Methods

### 2.1. Materials

Two complementary single-stranded 30-mer oligonucleotides: capture probe, 5′-GAGTAAAGTTAATACCTTTGCTCATTGACG-3′; and target probe, 3′-CTCATTTCAATTATGGAAACGAGTAACTGC-5′ were obtained from Integrated DNA Technologies, Inc. (Leuven, Belgium). Tyramine, *N*-hydroxysuccinimide (NHS), *N*-(3-dimethylaminopropyl) N-ethylcarbodiimide hydrochloride (EDC), 6-mercaptohexanol (MCH) and 1-dodecane thiol were obtained from Sigma-Aldrich (Steinheim, Germany), while sodium hydroxide and absolute ethanol (99.8%) were obtained from VWR international (Leuven, Belgium). Regeneration solution and buffers were prepared in ultrapure water (Millipore purification system, Bedford, MA, USA), filtered through a membrane with pores of 0.22 μm and degassed prior to use.

### 2.2. Methods

#### 2.2.1. Cleaning and Modification of Sensor Chips

In this study, the custom-made 3 mm diameter disposable gold electrodes from Academic workshop (Linköping University, Linköping, Sweden) were used as sensor chips. Before use, the sensor chips were cleaned in acetone, ethanol and finally in Piranha solution, as described elsewhere [6].

The modification with polytyramine film (Pty film) was made by coating the cleaned sensor chip with Pty film. The coating of the sensor chip was achieved by electropolymerization of tyramine on the sensor chip surface using cyclic voltammetry (CV) [10]. The coated sensor chip was then rinsed with ultrapure water to remove any loosely bound polymer.

The modification with 6-mercaptohexanol (MCH) was made by adsorption of surface assembled monolayers (SAMs) of MCH on the cleaned sensor chip. The sensor chip was dipped in 3.7 mM MCH solution (in 99.8% ethanol) for 1 h, followed by rinsing with ultrapure water to remove any loosely bound MCH [19].

#### 2.2.2. Immobilization of Capture Probe on the Modified Sensor Chips

Pty- and MCH-modified sensor chips were placed on a Petri dish. Then, 10 µL of a solution of 10 µM capture probe in 10 mM potassium phosphate buffer (PB), pH 7.2, containing 5 mM EDC and 8 mM NHS, was dropped on each sensor chip surface [23]. The sensor chips were then left at room temperature for 2 h.

The terminal phosphate group of the capture probe (ssDNA) was covalently coupled to either Pty film via primary amine group or MCH-SAM via hydroxyl group, on their respective modified sensor chip surfaces. The DNA phosphate group bound to primary amine and hydroxyl groups to form phosphoramide and phosphate ester bonds, respectively [19]. After immobilization, DNA-modified sensor chips were rinsed with ultrapure water, and finally the sensor chips were immersed in 1-dodecanethiol (10 mM in 99.8% ethanol) for 20 min in order to block pinholes on the affinity surface [5,6]. The modified sensor chips were stored at 4 °C until use.

**Figure 1:**
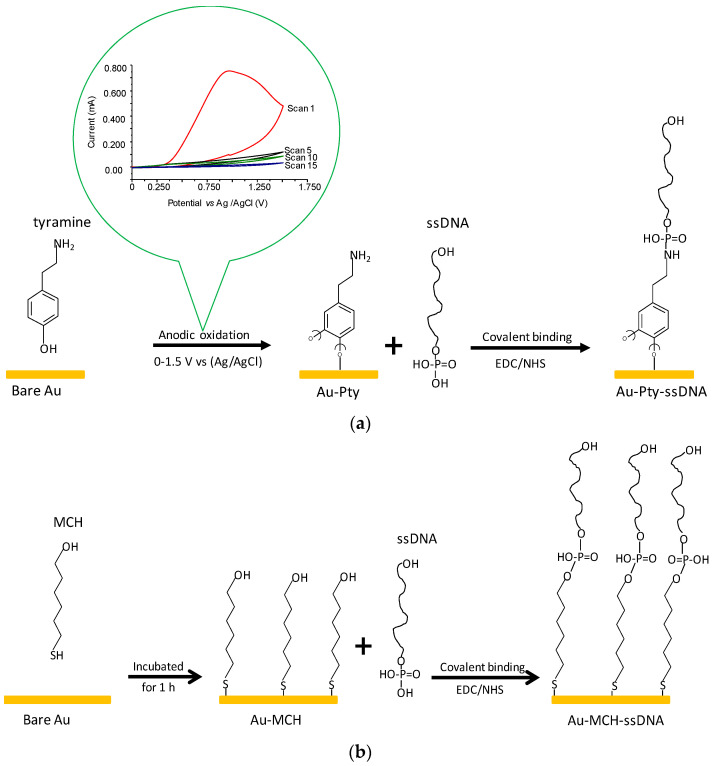
(**a**) Sensor chip surface modified with Pty film and followed by addition of ssDNA to form phosphoramide ester bond. The inset shows the reduction in anodic peak current during electropolymerization of tyramine on the sensor chip surface (scan numbers 1, 5, 10 and 15). (**b**) Sensor chip surface modified with MCH-SAM and followed by addition of ssDNA to form phosphate ester bond.

Figure 1a,b show the schematically summarized modification procedures of the Pty-film- and MCH-SAM-modified sensor chip surfaces, respectively.

#### 2.2.3. Electrochemical Characterizations

The surfaces of the sensor chips were electrochemically characterized, before and after chemical modifications, as well as after immobilization of ssDNA on the surfaces. The efficiency of the formation of modification layers on the sensor chip surfaces can be described in terms of surface coverage, which was estimated by comparing the areas of the gold oxide (AuO) reduction peaks and/or by calculating the difference in the electric charges exchanged during the reduction of AuO for the surfaces of the modified and bare gold sensor chips using CV in 0.1 M H_2_SO_4_ [24]. The exchanged electric charges were calculated using General Purpose Electrochemistry System (GPES) software (Metrohm Autolab, Utrecht, The Netherlands). The AuO reduction peaks for bare and modified gold sensor chips were integrated and the percentage coverage of a modified gold sensor chip surface was determined, as shown in Equation (1).

(1) $$\%\;\mathrm{Surface}\;\mathrm{coverage}=\frac{Q_{\mathrm{Bare}}-Q_{\mathrm{Mod}}}{Q_{\mathrm{Bare}}}$$

where Q_Bare_ and Q_Mod_ (C cm^−2^) are quantities of the exchanged electric charges in the reduction of AuO for bare and modified sensor chip surfaces, respectively.

In the analytical procedure, higher temperatures are desirable to enhance the selectivity of the DNA sensor. For that reason, a predictable pattern describing the capacitance change due to temperature elevation for an interface-modified sensor chip is essential. Therefore, the capacitances of the modified and bare sensor chips were studied at room temperature (RT, 23 °C) and at elevated temperature (50 °C) for 30 min using the CapSenze™ Biosystem (CapSenze Biosystem AB, Billeberga, Sweden).

One must keep in mind that the conditions mentioned for the analysis of DNA are suitable in laboratory studies, as described in this paper. In cases where protein is available, one may find disturbances due to the denaturation of proteins that do not stand the temperature increase. If heterogeneous biological media are analyzed, it might be recommendable to apply a pre-column for capturing the nucleic acids while all other molecules are removed. The nucleic acid can be released and then analyzed with the technology described above.

In earlier studies for the monitoring of proteins, it was clearly demonstrated that provided a capture molecule was present, then irrelevant molecules could be removed by a pulse of buffer before the assay [10].

#### 2.2.4. Atomic Force Microscopy

The surface topography of the non-modified and modified sensor chips in air was characterized by contact mode AFM at ambient temperature using a contact cantilever, PPP-CONSTCR (Park system, Suwon, Korea), with nominal resonance frequency of 23 kHz, thickness of 1 µm and force constant of 0.2 N m^−1^. Imaged area for each sample was 5 µm × 5 µm. However, the surface topography of the modified sensor chips that contained capture probe (ssDNA) was instead characterized by non-contact mode AFM, using a cantilever PPP-NHCR (Park system, Suwon, Korea), with thickness 4 µm, resonance frequency in air of 330 kHz and force constant 42 N m^−1^. In a mode with no direct contact AFM, the imaged area for each sample was 1 µm × 1 µm.

The following parameters (i)–(iv) were measured:

(i) Ra = average roughness
(ii) Rz = ten-point mean height roughness
(iii) Rq = root mean square roughness
(iv) Rpv = peak to valley roughness

A comprehensive analysis of sample surface properties was made using principal amplitude parameters such as the average roughness (Ra), ten-point mean height roughness (Rz), root mean square roughness (Rq) and peak to valley roughness (Rpv). The mentioned parameters quantify the surface properties and provide valuable insight regarding the quality of the layer on the surface [25].

#### 2.2.5. Capacitance Measurements

**Figure 2:**
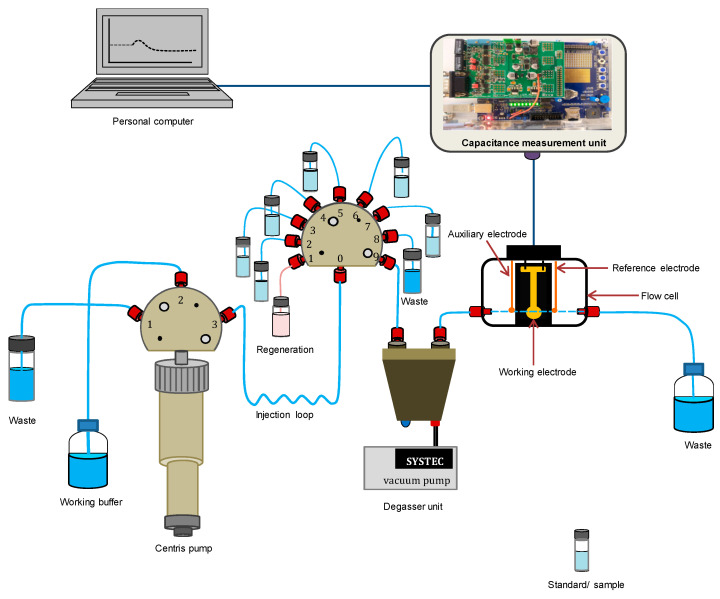
Schematic drawing of the flow-injection analytical system (CapSenze™ Biosystem).

The current pulse capacitance measurements were performed using an automated sequential injection flow system (CapSenze™ Biosystem), as seen in Figure 2.

The CapSenze™ Biosystem consists of the Cavro Centris pump (Tecan, Crailsheim, Germany) integrated with a 3-port valve (also Tecan), which in turn is connected to a 9-port valve via an injection loop. It also consists of a degasser unit, a three-electrode flow-cell, and a CapSenze capacitance measurement unit [22]. Port #1 of the 3-port valve is used as waste disposal during the initialization of the syringe pump. Port #2 is connected to the working buffer (PB) solution in which the syringe plunger will suck and eject the PB solution. The 9-port valve is used to automatically and serially inject 250 µL of a standard/sample and regeneration solutions into the flow cell via a degasser unit. The working buffer is continuously supplied to the flow cell by the syringe pump. The flow cell is connected to the capacitive biosensor, controlled by the software, and equipped with a resistor, which is connected between a potentiostat and the flow cell. Hence, an interrupted constant current pulse is supplied to the flow cell. The system has an inbuilt analog-to digital converter (ADC) unit. Therefore, the capacitance across the sensor chip/solution interface is automatically determined by the system from the slope of the potential curve (voltage vs. time) using Equation (2), as described elsewhere [22].

(2) $$C=\frac{Q}{V}=\frac{\mathrm{idt}}{\mathrm{dV}}=\frac{i\;}{u}$$

where C, Q and V are the total capacitance, accumulated charge and built-in potential across the sensor chip/solution interface, respectively; i is the electrical current supplied to the sensor chip; t is the current pulse period; u is the slope of the linear curve; and V is plotted against t. The total capacitance, C, across the sensor chip/solution interface is the sum of several capacitors in series Equation (3) [5,18].

(3) $$\frac{1}{C}=\frac{1}{C_{\mathrm{mat}}}+\frac{1}{C_{\mathrm{cap}}}+\frac{1}{C_{\mathrm{dl}}}$$

where C_mat_ is the capacitance of the insulating layer on the sensor chip surface, C_cap_ is the capture probe capacitance and C_dl_ is the capacitance of the diffusion layer.

When buffer is continuously pumped into the system and the current pulse is intermittently applied after every 60 s, the system will register a constant capacitance C, as a baseline. However, the introduction of a target probe on the sensor chip hosting capture probes will lead to a hybridization reaction, which in turn results in a decrease in C. This is due to the displacement of the counter ions from the electrode surface, as well as a reduction in the capture probe layer dielectric constant [5].

#### 2.2.6. Analytical Characterizations

Samples of the target probe were prepared in PB at concentrations 10^−13^, 10^−12^, 10^−11^, 10^−10^, 10^−9^, 10^−8^ and 10^−7^ M. Samples of each concentration were applied on the Pty-ssDNA- and MCH-ssDNA-modified sensor chips in triplicate. The relationship between registered capacitance change (ΔC) and the concentrations of the target probe was linear, and the limit of detections (LODs) of the Pty-ssDNA and MCH-ssDNA sensor chips were determined as described elsewhere [26].

For the selectivity study, the above experiments were repeated by injecting 10^−8^ M of non-target probe at 23 °C and 50 °C. Solutions of the capture probe were used as a non-target probe. The results were compared with those obtained from injecting the target complementary probe.

The sensor chips with different insulating layers were evaluated for re-usability by repetitively applying 10^−9^ M of the target probe followed by a regeneration step after each assay, up to 15 cycles.

Electropolymerization of tyramine was carried out on the gold sensor chips and the number of cycles of cyclic voltammetry was varied. The results of the analysis were evaluated when the sensor chip was used for analysis. However, there was no significant difference after 15 cycles.

## 3. Results and Discussion

### 3.1. Optimum Deposition Conditions

The factors affecting the deposition of the Pty and MCH immobilization layers on the sensor chip surfaces were previously optimized in order to maximize the immobilization of the capture probe for a better performance of the capacitive DNA sensor.

For the deposition of Pty film on the gold sensor chip, the effect of the number of cycles during the CV for the electropolymerization of tyramine was investigated. The insulation of the sensor chip surface was improved after between 5 to 15 cycles; furthermore, there was no significant difference after more than 15 cycles. Thus, 15 cycles was taken as the optimal number of cycles for the electropolymerization of Pty on the sensor chip surface. On the other hand, different incubation times and concentrations were investigated for the deposition of MCH on the sensor chip. The investigated concentrations of MCH were 3, 7, 10 and 40 mM. The deposition of MCH on the sensor chip surface was found to significantly increase with an increase in MCH concentrations for the first 15 min of incubation. However, there was no notable difference in surface coverage when the incubation time was prolonged to beyond 1 h. Additionally, MCH dissolved in 99.8% ethanol showed better insulation compared to that dissolved in ultrapure water. Therefore, 3.7 mM MCH dissolved in 99.8% ethanol and 1 h incubation time were selected as the optimum conditions for the insulation of the sensor chip surface with MCH-SAM.

### 3.2. Surface Coverage and Capacitance Baseline

Table 1 shows the percentages of surface coverage and capacitance baselines for Pty- and MCH-modified surfaces at their optimum experimental conditions, with and without ssDNA.

Pty film covered the sensor chip surface by 2.5 times more material than on the surface covered by MCH-SAM. Even after the immobilization of ssDNA on the Pty- and MCH-modified surfaces, the Pty-ssDNA displayed better insulating properties, with a surface coverage 2.5 times larger than MCH-ssDNA.

The recorded capacitance baseline of a bare gold sensor chip surface at RT was 10,420 nF cm^−2^. It was slightly increased to 10,790 nF cm^−2^ following the adsorption of Pty film on the sensor chip surface, suggesting that the Pty film, which is a non-conducting polymer layer, led to a considerable increase in the dielectric constant of the sensor chip surface. Another possible explanation is that the sensor chip surface became more hydrophilic after the electropolymerization of Pty film due to the Pty primary amine (-NH_2_) groups on the sensor chip surface; thus, the electrical double layer (EDL) at the surface solution interface is pulled much closer to the sensor chip surface, resulting in the observed increased capacitance.

Conversely, when MCH was deposited on the bare surface of the sensor chip, the capacitance decreased from 10,420 to 8510 nF cm^−2^. The decrease in capacitance is most likely due to the low dielectric constant [27] and the hydrophobic nature of the MCH-SAM [28]. The hydrophobicity of the MCH-SAM-modified surface is explained by the presence of the hydrophobic long hydrocarbon chains. Such a hydrophobic layer drives the electrical double layer (EDL) away from the surface of the sensor chip, hence resulting in a decrease in the capacitance baseline [5]. Ladik and co-workers [28] used contact-angle goniometry to investigate the hydrophobicity of MCH on the gold surface. Their results show that when only MCH was added on the gold slide the surface became more hydrophobic.

In all cases, the capacitances were observed to increase after the immobilization of ssDNA. This is in agreement with [28], that ssDNA is very hydrophilic, and as such its adsorption on the surface of the sensor chip improves the hydrophilicity of the surface, hence attracting the EDL closer to the surface of the sensor chip, which resulted in an increase in the capacitance baseline.

### 3.3. Electrochemical Properties

**Figure 3:**
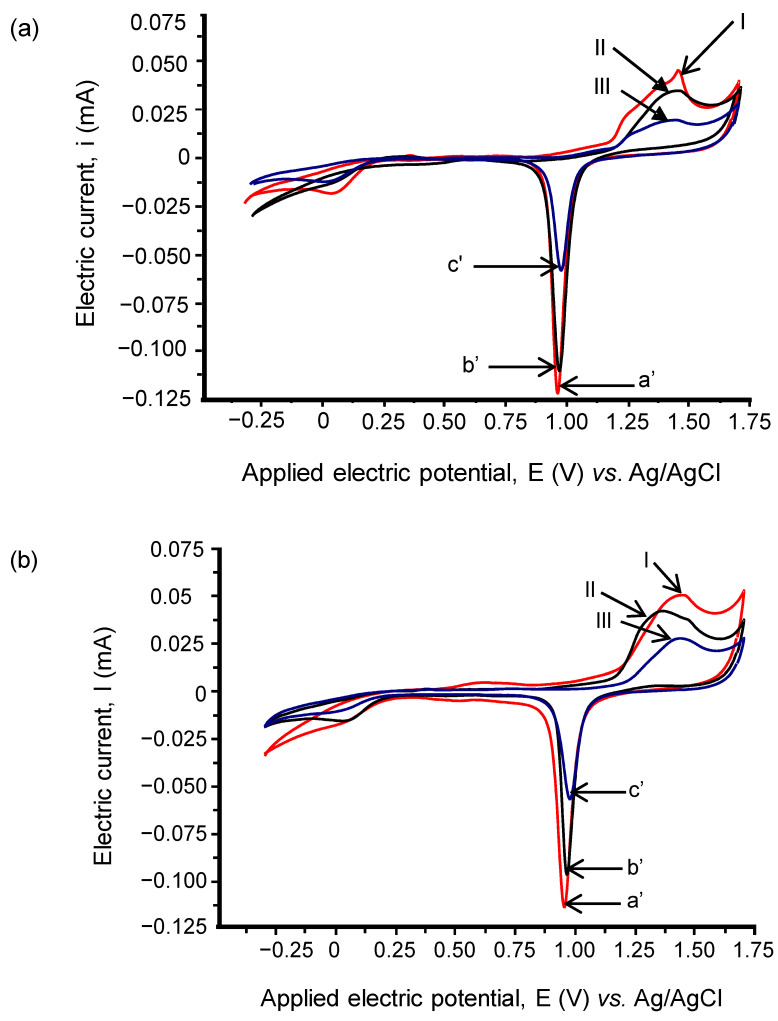
(**a**) Represents the reduction and oxidation peaks for: bare sensor chip, a′ and I; MCH-modified sensor chip, b′ and II; and Pty-modified sensor chip, c′ and III, respectively. (**b**) represents the reduction and oxidation peaks for: bare sensor chip, a′ and I; MCH-ssDNA-modified sensor chip, b′ and II; and Pty-ssDNA-modified sensor chip, c′ and III, respectively. The potential was swept in the range between −0.3 and 1.7 V (vs. Ag/ AgCl) in 100 mM H_2_SO_4_, at a sweep rate of 100 mVs^−1^.

The CVs for Pty-film- and MCH-SAM-modified sensor chips with and without ssDNA are compared with a bare sensor chip (Figure 3a,b).

In Figure 3a, the bare sensor chip surface displays the highest AuO reduction peak of all (voltammograms, a′). The deposition of MCH-SAM (voltammogram, b′) and Pty film (voltammogram, c′) on the sensor chip surfaces brought about a decrease in the AuO reduction peaks. The observation indicates that the presence of a Pty/MCH layer on the gold electrode surface prevents the oxidation of the gold electrode (see peaks I, II and III), resulting in a lower AuO reduction peak compared to the surface of the bare gold electrode. The magnitudes of the AuO reduction peaks followed the trend: bare sensor chip > MCH-SAM-modified sensor chip > Pty-film-modified sensor chip surface.

The observed magnitudes of the redox and AuO reduction peaks in Figure 3b followed the same trend as that observed in Figure 3a when the sensor chip surfaces were modified with MCH-SAM and Pty film only, i.e., bare > MCH-ssDNA-modified- > Pty-ssDNA-modified sensor chip surface.

The obtained electrochemical results demonstrate that the Pty film with or without ssDNA possess better insulating properties than MCH-SAM with or without ssDNA. This could be explained by the fact that, unlike MCH-SAM whose adsorption and stability on the surface is critically dependent upon the preparation and cleanliness of the surface [24], the electropolymerization of tyramine is not limited by the surface roughness [29]; hence, it forms a strong adhering film on the surface [30]. However, MCH offers advantages with respect to the ease of preparation and analysis.

### 3.4. Topographical Analysis

**Figure 4:**
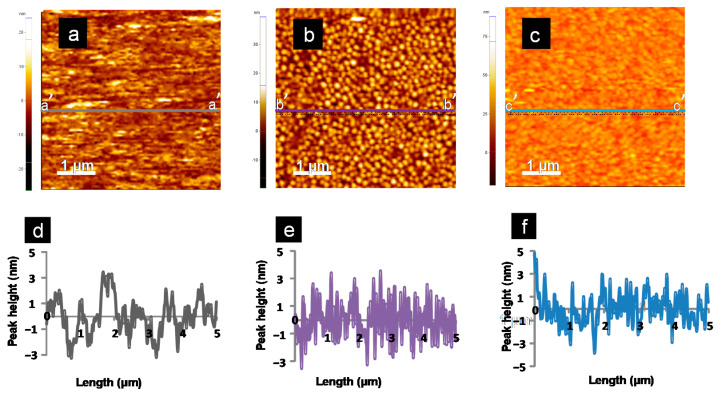
The 5 µm × 5 µm AFM images tapped in contact mode: (**a**) bare, (**b**) Pty-film-modified, and (**c**) MCH-SAM-modified gold sensor chips. The peak height profiles along the lines for (**a**–**c**) are shown in the figures marked (**d**–**f**), respectively.

The typical contact mode AFM images (scan size 5 µm × 5 µm) for the bare, Pty film and MCH-SAM gold sensor chip surfaces with their peak profiles are shown in Figure 4.

Topographic images showed the increase in roughness when sensor chip surfaces were modified with Pty film and MCH-SAM immobilization layers. The grain-like features with white heads and a dark brown color observed on the topographical images represent the peaks and depressions on the sensor chip surfaces, respectively. Therefore, the bare gold sensor chip surface is shown to be smoothest surface with the least number of peaks (Figure 4a) of all three, while the Pty-film-modified sensor chip surface (Figure 4b) is said to be a rougher surface, with more peaks than the MCH-modified sensor chip surface (Figure 4c).

The obtained information from topographical images is supported by the results obtained from peak height profiles analysis (Table 2).

Table 2 represents the surface peak profile analysis from Figure 4. Variations in the surface roughness, R_a_, and ten-point mean height roughness, R_z_, values have same trend as the variations in root mean square roughness, R_q_ values for bare, Pty and MCH gold sensor chip surfaces. The analysis showed the increase in roughness when the sensor-chip surface was modified with MCH-SAM and Pty film: the R_a_ value increased from 2.90 for bare sensor chip to 3.10 and 3.60 nm for MCH-SAM- and Pty-film-modified sensor chip surfaces, respectively. The observed increase in the roughness for Pty-film-modified sensor chip agrees well with the results obtained in the taping mode [8,10]. In addition, the R_q_ value for the bare sensor chip surface is relatively lower than that of MCH- and Pty-modified sensor chip surfaces, which indicates that the bare sensor chip surface was the smoothest surface of all.

Peak to valley height, R_pv_, is also considered as a very important parameter since it gives a good description of the overall roughness of the surface. It may be simply defined as a vertical distance from the highest peak on the profile to the lowest valley over the entire evaluation length of the profile [31]. Table 2 also shows that the R_pv_ values for the sensor chip surfaces are in the following order: bare < MCH < Pty. For high R_pv_ value, R_z_ is also high due to the strong dependence of R_z_ on the peak heights and valley depths. Therefore, the R_z_ values here indicate that the MCH-modified surface has less valley depth compared with the Pty-modified surface, but more than the bare sensor chip surface.

The results obtained from topographical images and peak height profile analysis prove the existence of the self-organization nature of SAM molecules (MCH), which produce a highly ordered smooth surface [32]. However, these findings suggest that Pty film is not as entirely self-limiting in growth as the electropolymerization of tyramine, resulting in a rougher surface than that formed by MCH-SAM.

**Figure 5:**
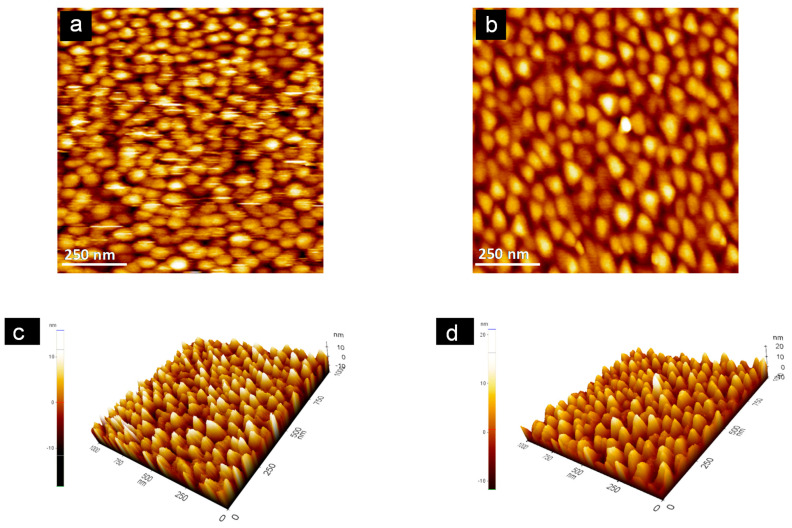
Non-contact mode AFM images (scan size 1 µm × 1 µm) of (**a**) gold sensor chip surface after being modified with Pty and coupled with ssDNA, and (**b**) gold sensor chip surface after being modified with MCH and coupled with ssDNA. The three-dimensional (3D) images of (**a**,**b**) are shown in the figures marked (**c**,**d**), respectively.

For the Pty-ssDNA-modified surface, there were more white spots observed on the surface (Figure 5a,c than in the MCH-ssDNA-modified surface (Figure 5b,d).

The white spots are supposed to be ssDNA molecule peaks. Therefore, the Pty-modified sensor chip surface is said to be more densely packed with capture probe ssDNA than the MCH-modified sensor chip surface. Pty film presents one reactive amine group per moiety which is easily protonated (–NH_3_^+^). Such a positively charged surface favors the immobilization of negatively charged molecules such as DNA. Hence, ssDNA molecules bearing negatively charged phosphate terminal functional groups were easily immobilized on the Pty film, resulting in a higher density of surface-bound capture probe ssDNA [33]. The observation was supported by the values of the roughness parameters obtained from peak height profile analysis, as shown in Table 2. The roughness factors of the Pty-modified sensor chip increased after the immobilization of capture probe ssDNA (Table 2). However, for the MCH-modified sensor chip, there was a decrease in surface roughness when capture probe ssDNA was immobilized onto it. One possible explanation could be that the capture probe ssDNA was non-specifically adsorbed onto uncovered spaces of the MCH-modified sensor chip surface, resulting in a decrease in surface roughness. The defects on the MCH-SAM sensor chip surface are mainly caused by a slow reorganization process. It usually requires some hours to maximize the molecules density and to reduce the defects on a sensor chip surface [32].

The AFM information indicates that Pty film offers a better sensor chip surface coverage with a higher capture probe surface density than MCH-SAM.

### 3.5. Sensitivity

**Figure 6:**
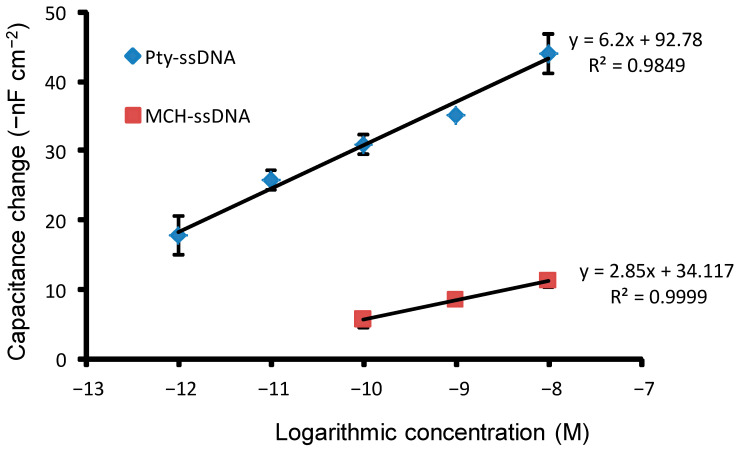
Calibration curves showing linearity range for target ssDNA hybridized with capture probe on the different sensor chips modified with Pty-ssDNA and MCH-ssDNA.

The calibration curves of the capacitive DNA sensor for Pty-ssDNA- and MCH-ssDNA modified sensor chips in response to the concentration of target complementary ssDNA are depicted in Figure 6.

The curves exhibited linear patterns at log concentrations, equivalent to molar concentrations in the ranges of: 10^−8^ to 10^−12^ M for Pty-ssDNA-modified surface and 10^−8^ to 10^−10^ M for MCH-ssDNA-modified surface, with LODs of 4.0 × 10^−13^ M and 7.0 × 10^−11^ M, respectively. The sensor chip surface and electrolyte interface (solution) act as two parallel plates in a capacitor, and the accumulated capacitance across the two plates is inversely proportional to the distance between the plates [5]. As such, when the distance between the sensor chip surface and the electrolyte interface increases, the total capacitance decreases. This decrease in capacitance is somewhat directly proportional to the applied molar concentrations of the complementary target DNA, 10^−8^ to 10^−12^ M and 10^−8^ to 10^−10^ M for Pty-ssDNA- and MCH-ssDNA-modified surfaces, respectively, as shown in Figure 6 above. At higher and lower molar concentration ranges, the relationship between the applied molar concentrations and the decrease in capacitance becomes nonlinear (not shown). This feature can be explained by the fact that, while at higher molar concentration, the availability of capture probe becomes a driving force of the reaction (hybridization), and thus the interaction between capture DNA probe with complementary target DNA becomes ‘complementary target DNA-dependent’; at lower molar concentrations, the availability of complementary target DNA becomes a driving force of the reaction (hybridization), and thus the interaction between capture DNA probe and complementary target DNA becomes ‘capture probe- dependent’; therefore, in both cases, the curves deviate from linearity.

The sensitivity and signal amplitude depend on the amount of the immobilized capture probe, ssDNA. At low concentrations, the number of captured target DNA molecules at equilibrium is proportional to the number of capture probe molecules [34]; thus, maximizing the amount of capture probe on the sensor chip is essential for ultrasensitivity. The presence of more ssDNA capture probe molecules on the sensor chip provides more biosensor hybridization sites for complementary target ssDNA molecules and hence improves the biosensor sensitivity. These results complement the previously obtained results from electrochemical and topographical analyses, which indicate that Pty film exhibited good insulating properties and provided many more spaces for the immobilization of the capture probe, which resulted in a higher capturing capacity. The higher capturing capacity led to the higher response signal and the lower LOD values, which are advantageous for capture assays involving small DNA molecules and for a sample with a very low concentration of target DNA molecules, respectively [34].

### 3.6. Selectivity

**Figure 7:**
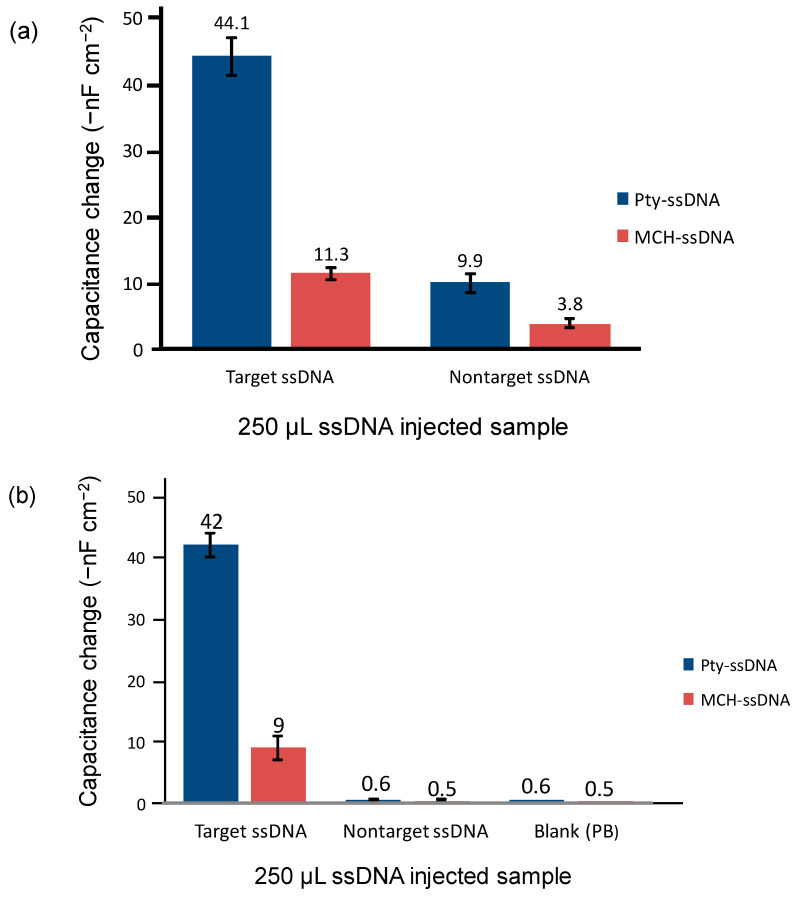
The amplitude of responses from Pty-ssDNA and MCH-ssDNA sensor chips to injection of equimolar concentrations, 10^−8^ M, of target and non-target ssDNA samples at (**a**) 23 °C (RT) and (**b**) 50 °C hybridization temperatures, respectively.

The selectivity of the sensor chips was evaluated at both room temperature (23 °C) and elevated temperature (50 °C), as shown in Figure 7a,b, respectively.

Although the response from the Pty-ssDNA-modified sensor chip as compared with that of the non-target ssDNA at 23 °C was much higher (9.9 ± 1.4 −nF cm^−2^) than that obtained for the MCH-ssDNA-modified sensor chip (3.8 ± 0.8 −nF cm^−2^), this non-target response is still 25% of a target response from the same sensor chip, whilst the non-target response from the MCH-ssDNA sensor chip accounts for 36% of its target response (Figure 7a). The Pty film has favorable permselective properties [35], thus, preventing interfering species from approaching or contaminating the sensor chip. Furthermore, an effective selectivity for both the MCH and Pty-ssDNA chips was obtained when the hybridization temperature was elevated to 50 °C (Figure 7b). The obtained response signals against the non-target sample were 0.6 ± 0.1 and 0.5 ± 0.2 −nF cm^−2^, for Pty-ssDNA and MCH-ssDNA sensor chips, respectively. These signals are regarded as false signals caused by a pressure drop (back-pressure) due to sample injection. The assumption was confirmed when the response signals obtained against blank (10 mM PB) samples corresponded with the signals against non-target samples. The obtained signals from blank samples were 0.6 and 0.5 −nF cm^−2^ for Pty-ssDNA and MCH-ssDNA sensor chips, respectively. The elevated temperature (50 °C) did not affect the signal amplitude from hybridization of the target probe on the Pty-ssDNA sensor chip; however, it did on the MCH-ssDNA sensor chip. The signal amplitude was significantly reduced almost by 20%, from 11 −nF cm^−2^ at RT to 9 −nF cm^−2^ at 50 °C. For the Pty-ssDNA-modified sensor chip, the reduced signal was less than 5%, from 44 −nF cm^−2^ at RT to 42 −nF cm^−2^.

The observed lower signal amplitude on the MCH-ssDNA sensor chip at 50 °C than at RT could be due to the rearrangement of the MCH-ssDNA layer during a baseline stabilization prior to analysis. This led to the decrease in the capacitance baseline in such a way that when the target probe was hybridized on the surface it could not produce the same signal response as that obtained at RT. On other hand, the obtained larger signal at RT was somewhat contributed to the non-specific adsorption of target ssDNA.

### 3.7. Re-Usability

**Figure 8:**
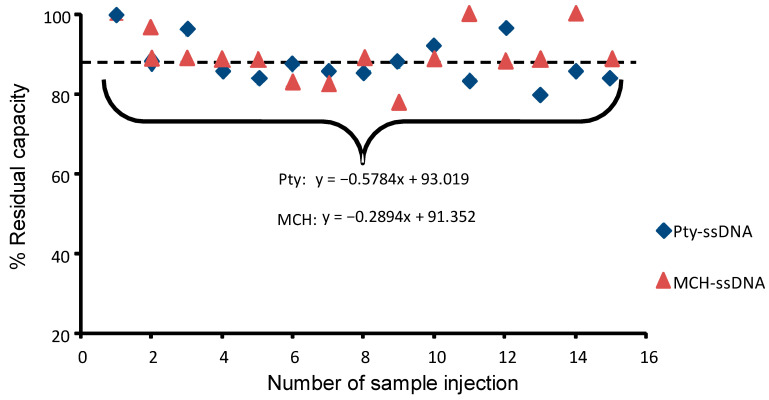
Reusability of Pty-ssDNA- and MCH-ssDNA-modified sensor chips. A total of 250 µL of a 10^−9^ M target ssDNA was applied 15 times on each sensor chip with a regeneration step between each individual assay. Symbols represent averages of triplicate data collected from CapSenze™ Biosystem.

High surface stability provides accuracy and precision, and thus allows repeated analysis on the same surface. Figure 8 shows the percentage residual capacity of the Pty-ssDNA and MCH-ssDNA sensors compared to the number of injections.

For 15 cycles, the binding capacity of Pty-ssDNA and MCH-ssDNA retained about 90% of the original capacitance change. Hence, both types of sensor chips can be re-used with good reproducibility more than 15 times, with relative standard deviation (% RSD) of 6%, and 7%, respectively. The low % RSD values suggest the high surface stability of the strongly electrodeposited Pty film and chemisorpted MCH-SAM on the sensor chip surfaces [32,36]. Similar results regarding reusability capacity, of about 12 to 20 times, for a Pty-ssDNA-modified sensor chip in capacitive biosensor measurements were previously recorded [6,37].

## 4. Conclusions

The study demonstrated some of the physical, electrochemical and analytical characteristics of the Pty film and MCH-SAM-modified gold sensor chips. The obtained electrochemical (CV) and physical (AFM) information corresponded well with the analytical characteristics observed from the CapSenze™ Biosystem.

This study provided us with valuable sound scientific evidence that Pty film is a relatively more suitable insulating layer for a gold sensor chip in capacitive DNA sensor measurements. Pty film evidently showed a good surface coverage, giving fine insulating properties that provide a relatively high density of immobilized capture probe (ssDNA) and relatively high sensitivity and signal amplitude. Additionally, the Pty film surface was observed to be much more stable, and thus provided accuracy and precision in measurements; as such, it allowed more than 15 repeated analyses on the same surface.

## Figures and Tables

**Table 1:** Pty-film- and MCH-SAM-modified sensor chip surfaces with their % coverage and capacitances.

Parameter(s)	Bare Sensor Chip	Pty-Only Sensor Chip	MCH-Only Sensor Chip	Pty-ssDNA Sensor Chip	MCH-ssDNA Sensor Chip
% surface coverage	0	65 ± 2	26 ± 2	75 ± 3	30 ± 2
Capacitance/baseline (nF cm^−2^)	10,420	10,790	8510	11,040	9140

**Table 2:** Roughness parameters of the sensor chip surfaces.

Surface	R_a_ (nm)	R_z_ (nm)	R_q_ (nm)	R_pv_ (nm)
Bare	2.90	14.50	3.35	16.00
Pty	3.60	20.40	4.60	25.10
MCH	3.10	17.50	3.85	21.70
Pty-ssDNA	3.80	14.00	4.60	18.30
MCH-ssDNA	2.50	11.30	3.20	15.70

## Data Availability

The study did not report any data.
